# Chronic Viral Hepatitis in the Italian Prison Setting: Prevalence, Outcomes, Literature Needs and Perspectives

**DOI:** 10.3390/healthcare9091186

**Published:** 2021-09-09

**Authors:** Vito Fiore, Andrea De Vito, Emanuele Pontali, Luciano Lucania, Giordano Madeddu, Sergio Babudieri

**Affiliations:** 1 Unit of Infectious Diseases, Department of Medical, Surgical and Experimental Sciences, University of Sassari, 07100 Sassari, Italy; andreadevitoaho@gmail.com (A.D.V.); giordano@uniss.it (G.M.); babuder@uniss.it (S.B.); 2 Infectious Disease Unit, Galliera Hospital, 16142 Genoa, Italy; pontals@yahoo.com; 3 Italian Society of Penitentiary Healthcare, 89121 Reggio Calabria, Italy; lucianolucania@virgilio.it

**Keywords:** penitentiary medicine, inmates and infectious diseases, prison environment

## Abstract

Compared with the general population, incarcerated people have a higher prevalence of several communicable diseases, including viral hepatitis. Nevertheless, there is still a lack of literature in field. Our review aims to shed the actual knowledge on viral hepatitis among incarcerated people in Italy. We performed a comprehensive literature search, through key electronic databases (Scopus, Pubmed–MEDLINE) and search engines (Google Scholar), of peer-reviewed publications (articles and reviews), grey literature on viral hepatitis prevalence, and models proposed for active case finding and control strategies in prison settings. We found that viral hepatitis epidemiology drastically changed in the last five years, particularly on hepatitis C virus (HCV), reporting an HCV antibody (HCV-Ab) prevalence decrease from up to 38% to ˂20% in penitentiary institutes, as well as an even more important reduction in active infections. Probably, the availability of direct-acting antivirals is contributing to this scenario. However, there is a lack of data available regarding incarcerated women. For this reason, more tailored interventions are needed for this sub-population. Judiciary and regulatory bodies should be prompted to discuss and define specific regulations to optimize case active finding strategies, guarantee wide access to effective preventive and treatment options for viral hepatitis and enhance treatment management.

## 1. Introduction

In 2018, ~500,000 people were detained in correctional facilities in the European Union (EU), with one incarcerated person every 896 people (111/100,000 people) [1].

Incarcerated people come often from underserved communities, including disadvantaged groups, migrants, and ethnical minorities backgrounds [2,3,4]. Moreover, prison populations mainly consist of marginalized individuals with lower healthcare provision and use before detention [5].

According to the United Nations Basic Principles, incarcerated people are considered people in the greatest need from a pure healthcare perspective [6]. Therefore, they ‘shall have access to the health services available in the country without discrimination on the grounds of their legal situation’ [6]. On this line, the ‘Dublin Declaration on HIV and AIDS in Europe and Central Asia’ states that a good prison health is a good public health [7]. However, incarcerated people still have limited healthcare provisions than free citizens in European countries [2,8].

Compared with the general population, incarcerated people have a higher prevalence of several communicable diseases, including human immunodeficiency virus (HIV), hepatitis B virus (HBV), hepatitis C virus (HCV), other sexually transmitted diseases (STDs), and tuberculosis (TB) [9,10]. Furthermore, they are exposed to an increased risk of acquiring such infections while in correctional facilities, and may contribute to the infections’ spread when coming back to freedom [8,10]. The high burden of infectious diseases in prison settings is exacerbated by limited access to prevention measures. Therefore, dynamic models are used to assess the spread and control of diseases within correctional facilities and their repercussions on the general population. According to such models, prison-based screening and treatment may be highly effective strategies for reducing the burden of blood-borne viruses (BBV), TB, and other STDs among incarcerated people and the general community [11].

The prison settings offer a great opportunity for primary, secondary, and tertiary prevention—if adequate care is provided—and early viral infection diagnosis opens the way to rapid and appropriate antiviral therapies for all incarcerated populations [8,12].

There is a large heterogeneity regarding communicable diseases prevalence and care in EU prison settings [13,14]. A detailed picture of the infections’ prevalence in different European Countries may be important to identify treatment gaps and predispose proper interventions. The availability of direct acting antivirals (DAAs), with high efficacy and a favorable toxicity profile would make possible HCV elimination plans in prison settings. However, clinical staging of all HCV viremic patients before or just after initiating therapy has to be planned [8].

On 28 February 2021, the 190 Penitentiary Institutes of the Italian correctional system hosted 53,697 incarcerated people. Of them, 2252 (4.2%) were women, 17,306 (32.2%) were foreigners, and the overcrowding was 6.2%. The 47% of detentions were related to drug offenses [15]. Among them, high-risk behaviors for BBV appear widespread, due to needle/sharps items/injection equipment exchange, unsterile tattooing, promiscuous and violent sexual relations, sharing of shaving razors inside the overcrowded cells, episodes of violence with wounds, and blood mingling.

Although infection management in Italian prisons could benefit from better understanding, to our knowledge, only scant information is available on the prevalence and management of viral hepatitis in this setting. Our review aims to shed actual knowledge on viral hepatitis among incarcerated people in Italy.

## 2. Methods

We performed a comprehensive literature search, through key electronic databases (Scopus, Pubmed—MEDLINE) and search engines (Google Scholar), of peer-reviewed publications (articles and reviews), grey literature on HBV and HCV prevalence, and models proposed for active case finding and control strategies in prison settings. Prison settings were defined as prisons, jails, and other custodial settings (excluding migrant facilities and police detention rooms). Prison population was defined as all incarcerated adult population (≥18 years).

The search strategy included the following terms: prison health, prison settings, and HCV or HBV and Italy, with no time limits or language restrictions. We screened the articles by title and abstract in full text, if relevant. To complement the evidence from the peer-reviewed literature, we searched for articles, abstracts, research reports, case studies, service models, and clinical protocols available on the web. Conference abstracts were checked for duplication with included peer-reviewed literature. In that case, the full-text article was chosen.

## 3. Results

### 3.1. Reported Prevalence of Blood-Borne Viruses in Correctional Facilities: Older Concepts

In 2005, Babudieri et al. conducted a cross-sectional study on the correlates of infection for HCV, HBV and HIV among incarcerated people from eight Italian prisons [16]. Overall, 973 inmates were enrolled. In this sample, high seroprevalence rates of HCV and HBV were reported (HCV: 38.0%; HBcAb: 52.7%; HBsAg: 6.7%). People who inject drugs (PWIDs) were more likely to test positive for HCV. HCV prevalence showed a U-shape (min = 31 years–max = 45 years), while HBsAg and HBcAb prevalence increased with age. Tattoos were associated with HCV positivity, while prison stay duration had a positive association with HBcAb positivity.

In a 2009, cross-sectional study involving 15,751 incarcerated people in Tuscany, Voller et al. reported a HCV and HBsAg prevalence of 9% and 2.2%, respectively [17]. Interestingly, this study showed a lower prevalence than the others for both HCV and HBV infection. However, data came from a single region, almost half of the incarcerated population were foreigners, and only current active infections were considered.

In another Italian study by Sagnelli et al., referring to nine prisons for a total of 3468 incarcerated people, HBsAg was detected in 4.4% out of 2265 and HCV positivity in 22.8% out of 2241 tested people [18]. Noteworthy, in this study, a screening based on peer-to-peer communication, followed by blood sampling voluntarily, was performed. This approach successfully improved patients’ participation, reaching ~60% of people in detention (range 37.3–95.2%) and obtaining extremely higher participation than the traditional approach (screening at prison entry).

Brandolini et al. carried out a single-centre cross-sectional study in Milan in 2013 [19]. Overall, 695 incarcerated people were enrolled. HCV seroprevalence was 22.4%, and 60 (38.4%) were people living with HIV (PLWH).

### 3.2. New Evidence in Viral Hepatitis Epidemiology

Viral hepatitis epidemiology drastically changed in the last five years, reporting an HCV-Ab prevalence ˂20% in penitentiary institutes.

In 2016, Foschi et al. documented an HCV seroprevalence of 9.8% among 468 incarcerated people in three major Italian correctional facilities. Of them, 6.6% were HBsAg positive and 3.2% HIV-Ab positive [20]. In the same year, Babudieri reported data on 5353 incarcerated people, showing HCV seroprevalence, HBsAg, and HIV positivity in 11.3%, 3.5%, and 3.7% of cases, respectively [21].

Masarone et al. reported data on 670 incarcerated people. Data were concordant to previous studies, reporting HCV-Ab positivity ~14% [22].

Fiore et al. carried out a multicenter study on testing and treatment models for HCV in prison settings. In this case, HCV-Ab prevalence was ~10%, and active infection in ~41% of cases. Among people with active infection, nobody had HBsAg positivity, and only 3.6% were PLWH [23,24].

Substantially, the extensive use of DAAs and a fast-track diagnostic approach with quick tests seemed to have a high impact on HCV epidemiology. Furthermore, it allowed an easier individuation of other BBV.

The overview of included studies on HCV and HBV epidemiology in prison population has been reported in Table 1.

### 3.3. Special Populations: Incarcerated People Living with HIV/HCV Co-Infection

HCV prevalence has been reported to be higher among PLWH than in other incarcerated people [20,24].

In a 30-month study published in 2008, the prevalence of HBV and/or HCV co-infection among 173 incarcerated PLWH was evaluated [25]. Beyond the 90% who tested posi-tive for HCV, 77.4% had past HBV infection, and active HBV co-infection was found in 6.7% of cases. HIV, HCV, and HBsAg positivity co-existence was seen in 6.1%. PWIDs were ~90% of incarcerated people, and ~1% were MSM.

In 2015, Monarca et al. conducted a multicenter, cross-sectional study, investigating and assessing HIV prevalence, and HIV-related medical activities in Italian correctional institutes, including 15,675 people [26]. Among 338 incarcerated PLWH, 23 (6.8%) and 189 (55.9%) had HBV and HCV co-infection, respectively.

Pontali et al. prospectively evaluated the prevalence of HBV and/or HCV co-infection among incarcerated PLWH in one correctional facility over 10 years [27]. Overall, 365 people were consecutively evaluated. HCV co-infection was observed in >80% of incarcerated PLWH. Past and active HBV infections were found in 71.6% and 7.1% of cases, respectively. HIV/HCV/HBV co-infection was reported in 6% of patients. PWIDs were the large majority (80.5%).

Sanarico et al. analyzed the correlates between infection and the molecular characterization of BBV infections among 69 incarcerated PLWH. Overall, most incarcerated PLWH (92.8%) showed evidence of past/active HBV and/or HCV infection. Prevalence of both HBV and HCV was 81.2%, with a higher prevalence of HCV infection reported among Italians and drug users [28].

A study conducted by Pontali et al. on incarcerated PLWH hospital admissions in Italy (*n* = 85), documented a HCV and HBV infection in 54.1% and 2.4% of cases, respectively [29]. Studies on viral hepatitis among PLWH have been summarized in Table 2.

These studies have multiple advantages. In fact, the large sample size and the extended testing approach change the perspective of healthcare provision and use in prison settings. Furthermore, generating high rates of awareness, the target moves from caring for the single individual to caring (and planning) for the entire population. Furthermore, the highly active antiretroviral therapy (HAART) substantially changed the HIV epidemiology in penitentiary settings, highlighting again the usefulness of ‘treatment as prevention’. In fact, data coming from the Italian Society of Penitentiary Healthcare (SIMSPe), confirmed a reduction of 6.6% in HIV prevalence in penitentiary settings over 15 years (2003–2015) [21].

### 3.4. Patients’ Clinical Features

The first available Italian data on clinical staging of HCV positive incarcerated people are from a study carried out in 2016 [21]. Despite there were single-center data, this allowed an initial assessment about the dimension of eligible patients to the new anti-HCV DAAs. Among the 137 evaluated HCV chronic infections, 11.0% showed a fibrosis score (FIB-4) indicative of advanced fibrosis (value > 3.25), while those without apparent liver damage (value < 1.45) were 55.9%. Among patients <40 years, 95.6% had not a FIB-4 indicative for advanced fibrosis. It seems interesting to remark that, at the time of the study, nobody among the HCV-RNA-undetectable patients showed liver cirrhosis, despite the presence of many PWIDs and non-alcoholic-steato-hepatitis (NASH) cases.

The recent literature has confirmed this datum, highlighting how HCV infected incarcerated people are generally ≤42 years old, and have low liver stiffness in the most of cases (range 64.2–96%) [20,23,24,30].

All these data highlight that incarcerated population is younger and with lower liver stiffness than the general community. In this environment, it would be useful to apply a strategy that would include rapid test and staging with short treatment schedules to quickly reach the microelimination and reduce the loss to follow-up.

### 3.5. HCV Treatment and Outcomes

The HCV treatment outcome among incarcerated patients drastically changed from interferon to DAAs era. In 2013, the cascade of care among incarcerated people receiving interferon for HCV, showed very low treatment rates, and success rates were even lower. Among 135 patients with active HCV infection, only 26% received treatment due to contraindications, refuse, transfer, or not being evaluated by the specialist. Among treated patients, <50% had successful treatment [24]. When coming to the DAAs era, positive treatment outcomes progressively increased in the last years. In a multicenter study from 2018, 142 patients were treated with DAAs, with sustained virological response (SVR) rates of 90.8% [31].

In 2020, Fiore et al. performed a case-control study between incarcerated patients and people in freedom. Patients in the two groups were homogeneous for six factors (age, sex, risk factor, liver fibrosis, co-infections, and genotype). The results of this study showed two important milestones in prison literature. First of all, SVR rates were not different between incarcerated and in freedom communities (*p* = 0.21). Furthermore, SVR rates among incarcerated people were ~96% [32]. A multicenter, prospective study on HCV epidemiology and DAAs cascade of care in Italy was recently published. Data were even superior, with SVR rates of 98% [23]. In this case, the added value was also related to educational programs and linkage to care in the case of prison release or transfer.

Furthermore, the fast-track diagnostic approach, associated with a wide use of DAAs in the last years, seemed to modify the HCV epidemiology, in the same way as HAART did with HIV in the recent past.

### 3.6. Future Perspectives: HCV Fast-Track Test-and-Treat Approach

One of the biggest limitations highlighted in all literature regarding viral hepatitis treatment in prison settings is prison release/transfer, as well as the lack of a clear model of micro-elimination pathway. These represented the most important barriers to overcome when coming to viral hepatitis treatment, with national data showing a loss to follow-up or unplanned treatments interruption up to 20% during interferon era [24]. When coming to DAAs, rates were reduced to ~6% [31].

In the last two years, quick tests were shown to fit better with such challenging settings, reducing time for diagnosis and allowing a quicker treatment initiation. Actually, results on unplanned interruptions and SVR rates have been far superior to the past, highly encouraging this type of approach. This is the reason why some authors are proposing rapid tests as a new standard of care when approaching chronic viral hepatitis in penitentiary institutes. It is also argued that point-of-care exams could contribute to reaching even faster test, staging, and treatment [22,23].

### 3.7. Female Incarcerated Population: The Harder-to-Reach-Population

When considering all the available literature in field, the most important limitation pointed out by studies on viral hepatitis is the low percentage of included women (range 2.1–6.9%). Women represent <5% of incarcerated population [33]. It follows this is an even harder-to-reach sub-population. A future step would be to provide a better insight on viral hepatitis among female incarcerated people to shed light on this partially hidden epidemic.

## 4. Discussion

Italian correctional facilities data from 2005 to 2021 show that HCV and HBV infections have an extensive diffusion among incarcerated people.

This highlights how much this environment is fundamental in micro-elimination pathways. A high gap with the estimated prevalence in the general Italian population has been shown (HCV-Ab, 3.2%; HBsAg, 1.5%).

The high spread of viral hepatitis in the Italian prison settings, as already reported in other international studies, is related to several risk factors, which increase the infectious diseases transmission, such as low educational level, risky behaviors, social factors, and structural issues (e.g., overcrowding, lower hygiene) [12,33,34]. In our review, we observed that HBV and HCV infections are highly prevalent in subjects co-infected with HIV, IDUs, MSM with high-risk sexual behavior, and subjects who underwent unsterile tattooing or piercing. However, further studies are needed to assess the association between risk factors and different types of infections. Moreover, it is important to investigate how to identify these risk factors among prison inmates better and implement effective preventive and corrective actions and harm reduction strategies. Indeed, the peculiar characteristics of the prison setting and the difficulties in communication with inmates hinder the identification and prevention of these risk factors.

The high disease detection rate encountered in penitentiaries advocates for a strengthening of active case finding initiatives as a public health intervention. High disease prevalence represents a health issue not only for prisoners and people working inside prisons, but also for the general population, given that most of the inmates return to their communities after a short period of detention [35]. Active case finding is crucial to prevent further transmission of the disease, implement preventive measures, and start/continue treatment if needed. This activity is particularly important for diseases for which preventive or treatment options are available, like HBV and HCV. Among the possible preventive measures, needle exchange programs, opioid substitution therapy, condom distribution, and provision of sterile tattooing equipment should be considered. Unfortunately, the implementation of condom distribution and needle and syringe programs, despite being low-cost interventions, is still limited in Italy due to legal barriers.

In the case of HBV, a highly effective vaccine is available to prevent the infection spread. Therefore, it is crucial to implement a systematic hepatitis vaccination program for inmates and employees, together with counseling and clinical evaluation. The 17.8% of the Italian prison population is under 30 years of age and should have been vaccinated for HBV [34]. However, ~30% of incarcerated people are foreigners, often coming from countries where this vaccination is not included in the local schedules. Among them, serological status should be quickly assessed at entry to provide HBV vaccination to foreign inmates and older inmates at their first admission to prison [34]. Moreover, effective treatment options have been developed and well-studied in their safety profile for HBV chronically ill patients [27,36].

PLWH are more likely to present HCV and HBV co-infection when compared with other incarcerated people. This underlines the importance of a comprehensive screening approach as per WHO guidelines [27].

As published studies highlighted, treating HCV infection in prisons embodies the concept of ‘treatment as prevention’, reducing the possibility of new infections, developing advanced disease, and breaking the chain of transmission within both the prison system and the community [37].

The introduction of DAAs deeply changed HCV patients’ treatment and clearly improved treatment outcomes in prison settings. Second-generation DAAs present various advantages compared to peg-interferon therapies and first-generation DAAs, whose efficacy was limited by the high risk of toxicity, lower efficacy and prolonged therapy duration. In addition, new DAAs are characterized by markedly increased SVR rates, together with short schedules and low adverse reaction occurrence.

The young age and the low level of hepatic fibrosis usually reported among inmates further enhance the probabilities of achieving the highest rates of viral eradication in this setting. This is an important improvement considering that the chances of completing long therapies in prison are usually low due to legal and logistical issues [8].

Moreover, while peg-interferon is contraindicated in many prisoners (subjects with psychiatric disorders, drug/alcohol addiction, or tuberculosis infection), DAAs can be used in a much larger population and are more effective in PLWH [8]. Finally, DAAs can also be administered in combination with several other drugs without clinically significant interactions.

Hence, DAAs should be started as soon as possible in all HCV viremic patients to limit the infection spread both inside the penitentiary and to the external community [8,32]. Given the compliance to treatment is the key for treatment success, it is important to reduce transfers or releases of patients while on therapy, and therapeutic continuity should also be promoted after release through appropriate educational activities and linkages with external treatment centers [8,23].

## 5. Conclusions

Chronic viral hepatitis still represents one of the main health issues in Italian correctional facilities. The opportunity for appropriate case finding and treatment strategies in prison settings advocates for a deep revision of the relevant regulations to ensure optimal identification, prevention, and treatment for infectious diseases in correctional facilities.

Judiciary and regulatory bodies should be prompted to discuss and define specific regulations to optimize case active finding strategies, guarantee wide access to effective preventive and treatment options for HBV and HCV and enhance treatment management, ensuring therapeutic continuity both during detention and after release [8]. These strategies are important not only for prison health but also to reduce disease transmission, improving public health. This will be the next step towards viral hepatitis elimination, reaching 2030 World Health Organization targets [38].

## 6. Limitations of the Study

There is a lack of literature regarding models on viral hepatitis test and treatment in Italian literature. Furthermore, all studies showed how the approach is not homogeneous in all 190 national penitentiary institutes.

Our review included a low number of papers, and the most part were single-center studies. Furthermore, very limited data on female prison population were available. However, data were in line with the literature coming from other European Countries.

## Figures and Tables

**Table 1:** Overview on viral hepatitis epidemiology reports (values calculated from available data).

Literature Data	Year	Included Subjects	Median Age	Male Sex %	HCV-Ab Prevalence %	HBsAg/ HBcAb Prevalence	PWIDs	HIV-Ab +n (%)
Babudieri et al. [16]	2005	973	36	87%	38%	6.7%/59.4%	30.4%	73 (7.5)
Pontali et al. [25]	2008	173	NA	100%	90.8%	6.7%/77.4%	91.9%	173
Voller et al. [17]	2011	15,751	39.6	94.2%	9.0%	2.2%/NA	NA	NA
Sagnelli et al. [18]	2012	3468	NA	NA	22.8%	4.4%/NA	NA	89 (3.8)
Brandolini et al.	2013	695	43	97.8%	22.4%	6.2%/NA	NA	67 (9.6)
Foschi et al. [20]	2016	468	NA	NA	9.8%	6.6%/NA	NA	15 (3.2)
Babudieri [21]	2016	5353	NA	NA	11.3%	3.5%/NA	23,7%	200 (3.7)
Masarone et al. [22]	2020	670	NA	94.02%	14.05%	NA/NA	18.6%	NA
Fiore et al. [23]	2021	2376	42	98%	10.4%	-/NA	22.6%	3 (3.6)

PWIDs: people who inject drugs.

**Table 2:** Overview on viral hepatitis among people living with HIV (values calculated from available data).

Literature Data	Year	Included PLWH	Age, Median (IQR)	Male Sex	MSM	PWIDs	HCV Co-Infection	HBV Co-Infection	HIV/HCV/HBVCo-Infection
Pontali et al. [25]	2008	173	NA	100%	1.2%	91.9%	90.8%	6.7%	6.1
Monarca et al. [26]	2015	338	NA	97.6%	4.7%	51.7%	55.9%	6.8%	NA
Pontali et al. [27]	2016	365	NA	100%	1.8%	80.5%	82.7%	7.1%	6%
Sanarico et al. [28]	2016	69	43 (31–67)	95.6%	2.9%	78.3%	81.2%	81.2%	69.6%
Pontali et al. [29]	2017	85	48 (NA-NA)	96.5%	NA	11.8%	54.1%	2.4%	NA

PLWH: people living with HIV; MSM: men who have sex with men; PWIDs: people who inject drugs.

## Data Availability

The data analyzed during the current study are available from the corresponding author on reasonable request.
